# Examining the length of hospital stay and associated factors in adult patients with schizophrenia

**DOI:** 10.4102/sajpsychiatry.v32i0.2622

**Published:** 2026-04-08

**Authors:** Kearabilwe Mahlabane, Nabila Veyej, Tejil Morar

**Affiliations:** 1 Department of Psychiatry, Faculty of Health Sciences, University of the Witwatersrand, Johannesburg, South Africa

**Keywords:** length of stay, schizophrenia, South Africa, inpatient, prolonged stay, mental health services

## Abstract

**Background:**

Inpatient care is a key driver of mental healthcare costs in South Africa (SA). Schizophrenia is a common psychiatric diagnosis amongst hospitalised patients in the public sector of SA. Determining the length of stay (LOS) and associated factors in patients with schizophrenia could assist in effective utilisation of the mental healthcare budget.

**Aim:**

To determine the LOS and any associated socio-demographic and clinical factors in patients with schizophrenia.

**Setting:**

A psychiatric unit in a central public hospital, Chris Hani Baragwanath Academic Hospital (CHBAH) and a specialised psychiatric hospital, Tara H Moross (TARA) in Gauteng, SA.

**Methods:**

A quantitative retrospective review of the records of patients with schizophrenia admitted to CHBAH and discharged from CHBAH and TARA hospital over the study period was conducted. Information on the LOS, socio-demographic and clinical factors was collected upon discharge.

**Results:**

The median LOS for the 132 patients with schizophrenia was 42 days, with a mean of 55 days. In bivariate analysis, statistically significant factors associated with LOS were living arrangements (*p* = 0.036), number of previous admissions (first admission) (*p* = 0.009) and being on clozapine (*p* < 0.001). However, in regression analyses, only living alone remained statistically significant and associated with a shorter LOS (*p* = 0.004).

**Conclusion:**

Prolonged LOS indicates ongoing reliance on inpatient care due to service level constraints, including poorly resourced community psychiatric services and individual contextual factors. Both socio-demographic and clinical factors influenced LOS. Associated factors such as living alone and early clozapine initiation provide targets from which to start addressing modifiable contributors that may reduce LOS.

**Contribution:**

This study contributes to the limited data on the LOS for patients with schizophrenia in SA.

## Introduction

The length of stay (LOS) is an important indicator of healthcare delivery.^1^ It indicates the number of days between an individual patient’s admission and discharge.^2^ Length of stay guides service planning, resource distribution and carries major financial implications, driven by long hospital stays.^1^ This is relevant especially in South Africa (SA), where a mere 5% of the public health budget is allocated to mental health. Of this, a disproportionate amount of 86% is allocated to inpatient mental health care.^3^ Therefore, given a limited budget, optimising clinical care is a key focus to effectively manage LOS.^4^

The public sector in SA is responsible for servicing the mental healthcare requirements of 80% of the population. It is organised into levels of increasingly specialised care, from primary healthcare clinics to district, regional, central and specialised psychiatric hospitals, with patients routinely transitioning between these levels of care.^3,5^ Accordingly, inpatients requiring prolonged general hospital stay or those who cannot be safely managed in general hospital psychiatric units are transferred to specialised psychiatric hospitals, depending on bed availability at the psychiatric hospital.

The limited inpatient capacity for mental healthcare users is one of the reasons for the global shift towards deinstitutionalisation.^6^ However, progress regarding decentralisation of care in SA and other lower-to-middle-income countries (LMICs) has been slow because of poorly developed community psychiatric services, resulting in hospital expenditure still utilising most of the mental health budget.^3,5,7^ For example, in the years 2016–2017, the inpatient cost of mental health services was 2.25 billion South African rand (ZAR) compared to 276 million ZAR for outpatient mental health services in Gauteng.^3^ Therefore, in the public sector of SA, LOS is a useful measure of resource efficiency. This trend is also noted internationally, in the United States (US), where the estimated economic burden of schizophrenia doubled from an equivalent value of 1.5 trillion ZAR in 2013 to 4.9 trillion ZAR by 2019.^8^

A previous study from Gauteng revealed that schizophrenia, a chronic and functionally debilitating illness characterised by frequent relapses, is a common diagnosis that comprise 20% of all patients admitted to general hospital psychiatric units.^9^ Hence, information on LOS and its predictors in patients with schizophrenia is necessary to optimise inpatient care and reduce direct (treatment expense-related) as well as indirect costs. Indirect costs are not only related to opportunity and loss of income amongst patients, but also from the caregiver’s loss of earning potential, which is affected by poor work attendance, missed employment opportunities due to caregiving responsibilities, and the diversion of household resources spent on travelling to healthcare facilities and other expenses needed by the patient whilst admitted.^10,11^

Internationally, there is marked variability in the LOS for patients with schizophrenia at a general hospital, ranging from 9 days in the United States^8^ to 290 days in Japan^12^ because of differences in socio-demographic, clinical and treatment-related factors.^13,14^ The national average LOS for psychiatric admissions in SA ranges from 8.6 days in district hospitals to 157.1 days at specialised psychiatric hospitals.^3^ There is limited information on the LOS for patients with schizophrenia in SA. A study at a psychiatric hospital in Gauteng reported that the mean LOS for patients with schizophrenia and schizoaffective disorder was 128 days (standard deviation [s.d.] = 120.89).^15^ This prolonged LOS is in keeping with the level of hospital care, in that this is a specialised public health sector psychiatric hospital, and serves as a referral hospital for patients from acute psychiatric units with a primary diagnosis of a serious mental illness requiring medium to long-term hospitalisation. Another study at a general hospital psychiatric unit in Pretoria reported that 72% of the patients diagnosed with acute psychosis in their study had a LOS of more than 21 days. This study did not report the median or mean LOS.^2^

To the best of our knowledge, there is no information on the total LOS of patients with schizophrenia, from the initial admission to a general hospital to discharge from that general hospital or from a specialised psychiatric hospital in SA. The authors hypothesised that a combination of factors would influence LOS, and the LOS would be intermediate, falling between the shorter LOS in general hospitals and the longer LOS reported in the specialist psychiatric hospital. Furthermore, the international literature is inconsistent, with significant regional differences regarding the LOS and the associated socio-demographic and clinical factors in patients with schizophrenia.^13^

### Aim and objectives

This study aimed to examine the LOS in patients with schizophrenia who were admitted to CHBAH and discharged from CHBAH or a specialised psychiatric hospital in Gauteng, and to identify any socio-demographic and clinical factors associated with the LOS.

The study objectives were to:

- Describe the socio-demographic characteristics of the study population.
- Describe the clinical characteristics of the study population.
- Determine the LOS of patients with schizophrenia over the study period.
- Determine which, if any, socio-demographic and clinical factors are associated with LOS in patients with schizophrenia.

## Research methods and design

### Study design

This was a quantitative retrospective review of the medical records of adult patients with schizophrenia who were admitted to CHBAH and subsequently discharged from either CHBAH or from TARA hospital following a transfer from CHBAH.

### Setting

The study was conducted in the Department of Psychiatry at Chris Hani Baragwanath Academic Hospital in Soweto a large, urban township made up of informal and formal neighbourhoods ranging from working to middle-class profiles in the city of Johannesburg, SA. It is the city’s most populated area and has one of the highest unemployment rates. Literacy levels in the area are generally lower, given the area’s historically low education baseline.^16^

CHBAH is a central hospital with 155 beds that provides mental health services to individuals living in the catchment area of the hospital and supports care at district hospitals and primary healthcare clinics in the surrounding areas. Patients with schizophrenia who require longer-term treatment, or who cannot be managed at this facility, are transferred to any of the two specialised psychiatric hospital in Gauteng, namely TARA hospital and Sterkfontein psychiatric hospital. The clinical records of patients who were transferred from CHBAH to TARA hospital in the region were included in this study.

### Study population and sampling strategy

This was a non-probability convenience sample. The clinical records of patients 18 years and older with a Diagnostic and Statistical Manual of Mental Disorders, Fifth Edition (DSM 5) diagnosis of schizophrenia who were admitted to CHBAH between 01 January 2021 and 01 January 2023 and who were subsequently discharged from CHBAH or transferred to and discharged from one of the two specialised psychiatric hospitals located in the region during this period were included in the study. The records of patients who had a final discharge diagnosis other than schizophrenia, discharged from medical and/or surgical wards in CHBAH or the other specialised psychiatric hospitals in the region, were excluded from the analysis. Patients who were admitted on more than one occasion to CHBAH were excluded from the sample, and those still admitted at the time of data collection were also excluded, to avoid bias in the analysis of the association between LOS and socio-demographic and clinical factors.

A sample size of 132 was needed to provide sufficient power to determine associations. This was calculated based on a 95% confidence level, margin of error of 5.66, and expected s.d. of 32.75, using the range of average LOS (36.0–157.1 days) for psychiatric admissions at a central hospital and specialised psychiatric hospital in SA from an economic evaluation study combined with a national survey quantifying public expenditure on mental health^3^; the calculation employed *t*-distribution.

### Data collection

In this study, LOS was defined as the number of days a patient stayed in the hospital from the day of admission to the day of discharge. Chris Hani Baragwanath Academic Hospital maintains an electronic database of the discharge summaries of all patients admitted to the unit. Information regarding the LOS, socio-demographic and clinical characteristics was extracted by the primary researcher, from this database using a structured data collection sheet developed by the researchers, over a two month period. Hard copies of the discharge summaries of patients transferred from CHBAH to TARA hospital were collected and reviewed by the primary investigator as the site does not have an electronic database. The information was then collated onto the data collection sheet. All the data were then entered on a password-protected Microsoft Excel file by the primary researcher.

### Data analysis

Statistical analyses were conducted using R software (version 4.0.0; www.R-project.org).

Data were presented in tables and in text format. To describe the socio-demographic and clinical characteristics of the study population, continuous data were reported as medians and interquartile ranges (IQR) (for non-parametric data) or mean and s.d. (parametric data), and categorical data were reported as counts and percentages. To calculate the LOS of patients with schizophrenia over the study period, the data were reported as median and IQRs.

To determine the association between LOS of patients with schizophrenia and their socio-demographic and clinical characteristics, the data were first assessed for departure from normality using Shapiro–Wilk tests and visually inspected using quantile-quantile (QQ) plots. The data were non-parametric; therefore, the association between LOS and categorical variables was analysed using Mann–Whitney U tests (*k* = 2 levels) or Kruskal–Wallis tests (*k* > 2 levels).

Pairwise post hoc tests (with Bonferroni adjustments) were conducted following significant Kruskal–Wallis tests. Variables with counts below five were excluded from the analysis.

Variables found to be significant in bivariate analyses were further analysed to adjust for potential confounding. The outcome variable, LOS, demonstrated a significant departure from normality (*W* = 0.66, *p* < 0.001), with skewed residuals observed in linear regression. Thus, a generalised linear model (GLM) with a Gamma distribution and a log link function was employed to model LOS. Model estimates are reported as log-scale coefficients and exponentiated to yield odds ratios (exp(β)), which represent the multiplicative change in the expected LOS. Model significance was set at 0.05, and the tests were two-tailed.

### Ethical considerations

Ethical clearance to conduct this study was obtained from the University of the Witwatersrand Human Research Ethics Committee. (No. M240544 M240725-A-0002). Due to this being a retrospective review, informed consent was not required. Patient confidentiality was maintained by anonymising data using study numbers. The dataset was stored securely on a password-protected Microsoft Excel file and was only accessible to the primary researcher.

## Results

### Socio-demographic and clinical characteristics

Table 1 shows that the mean age of the 132 patients in this study was 34.9 years (s.d. = 12.1 years). Most patients were male, 78.8% (*n* = 104), single, 93.9% (*n* = 124), living with family members, 82.6% (*n* = 109) and unemployed, 90.2% (*n* = 119).

**TABLE 1:** Socio-demographic and clinical characteristics of study participants (*N* = 132).

Variables	Count (*n*)	%
**Gender**		
Male	104	78.8
Female	28	21.2
**Relationship status**		
Single and/or divorced	124	93.9
Married and/or in a stable relationship	7	5.3
Unknown	1	0.8
**Living arrangements**		
With family	109	82.6
Homeless	1	0.8
Alone	5	3.8
Institution	1	0.8
Unknown	16	12.1
**Employment status**		
Unemployed	119	90.2
Employed (permanent and/or temporary)	5	3.8
Student	8	6.1
**Duration of illness (years)**		
< 5	60	45.5
5–10	29	22.0
> 10	35	26.5
Unknown	8	6.1
**Number of previous admissions**		
First admission	34	25.8
Two or more	80	60.6
Unknown	18	13.6
**Schizophrenia symptom domains**		
Positive	106	80.3
Negative	8	6.1
Mixed	15	11.4
Catatonia	3	2.3
**Discharge hospital**		
Chris Hani Baragwanath Academic Hospital	119	90.2
TARA Hospital	13	9.8
**Discharge plan**		
Home	124	93.9
Placement and/or Non-governmental organisation	8	6.1
**Comorbid psychiatric illnesses**		
Yes	95	72.0
No	37	28.0
**Substance use disorder**	84	63.6
Polysubstance use (≥2)	29	34.5
Single-substance use	55	64.5
**Type of substance used**		
Cannabis	73	55.3
Alcohol	22	16.7
Opioid	3	2.3
Sedative	1	0.8
Stimulant	22	16.7
**Comorbid physical illness**		
Yes	37	28.0
No	95	72.0
**Other psychotropic medication**		
Mood stabilisers	26	19.7
Antidepressants	16	12.1
Anticholinergics	10	7.6

Note: Age – Mean age 34.9 years (s.d. = 12.1).

TARA, Tara H Moross Hospital.

Regarding the clinical characteristics, 45.5% (*n* = 60) of patients had a duration of illness of less than 5 years, 60.6% (*n* = 80) had two or more previous admissions to the central hospital, 80.3% (*n* = 106) had prominent positive symptoms on admission, 63.6% (*n* = 84) had a comorbid substance use disorder and 90.2% (*n* = 119) were discharged home from CHBAH. Olanzapine was the most frequently prescribed oral antipsychotic drug, 49.2% (*n* = 65), and about one quarter of the patients, 23.5% (*n* = 31), were on long-acting injectable antipsychotics (LAI-APs). Other classes of psychiatric medications were prescribed less often. These included mood stabilisers 19.7% (*n* = 26), antidepressant 12.1% (*n* = 16) and anticholinergic medications 7.6% (*n* = 10).

### The length of stay and the socio-demographic and clinical factors associated with length of stay

The median total LOS of the patients with schizophrenia admitted to CHBAH and discharged from CHBAH or TARA hospital was 42 days (IQR = 27 days, 95% confidence interval [CI] = 40.7–59.0 days), with a mean of 55.4 days (s.d. 55.1 years).

The mean LOS for those patients admitted to CHBAH and discharged from CHBAH was 42.7 days (s.d. 27.4 years). The mean LOS for patients who were admitted to CHBAH and required a transfer to TARA and then discharged from TARA hospital was 173.6 days (s.d. 96.9 years).

In bivariate analysis, Table 2 and Table 3 show that there was a statistically significant association between the LOS and living arrangements (*p* = 0.036), number of previous admissions (first admission) to CHBAH (*p* = 0.009) and the prescription of clozapine (*p* < 0.001). Patients who lived alone or had two or more previous admissions had a significantly shorter LOS compared to patients who lived with family and had one previous admission, whilst patients who were on clozapine had a significantly longer LOS compared to patients on other antipsychotics. Length of stay did not differ between patients with or without medical comorbidities, nor between those treated with antipsychotic monotherapy or polypharmacy. Similarly, LOS was comparable between single-substance and polysubstance users. The four antipsychotic or LAI treatment combinations also showed no significant differences in LOS, although the clozapine and LAI subgroup had a higher median LOS, which should be interpreted cautiously given the very small sample sizes of the LAI subgroups. Finally, the LOS was very similar for patients with any psychiatric comorbidity and those with none.

**TABLE 2:** Antipsychotic prescribing patterns.

Antipsychotic regimen	Count (*n*)	%
**Number of antipsychotics**		
Monotherapy	93.0	70.5
Polypharmacy	39.0	29.5
**LAI use**		
LAI only	2.0	1.5
LAI and clozapine	4.0	3.0
LAI and non-clozapine	25.0	18.9
No LAI	101.0	76.5
**Oral antipsychotics prescribed**		
Olanzapine	65.0	49.2
Amisulpride	16.0	12.1
Clozapine	15.0	11.3
Haloperidol	16.0	12.1
Quetiapine	4.0	3.0
Risperidone	23.0	17.4
**LAI-antipsychotics prescribed**		
Zuclopenthixol decanoate	3.0	2.3
Flupentixol decanoate	27.0	20.5
Paliperidone palmitate	1.0	0.8

LAI, long-acting injectable.

**TABLE 3:** Bivariate analysis of the association between the length of stay and socio-demographic and clinical characteristics.

Variables	Median	IQR	Statistics
Length of stay (days)	42.0	27.0	-
Age			*r_s_* = -0.05, *p* = 0.587
**Gender**			*W* = 1686.5, *p* = 0.201
Female	45.0	59.0	-
Male	42.0	39.0	-
**Relationship status**			*U* = 285.5, *p* = 0.131
Married and/or in a stable relationship	33.5	20.0	-
Single and/or divorced	42.0	39.0	-
**Living arrangements**			KW = 6.97, df = 2, *p* = 0.036
Living alone	17.0	23.0	-
Living with family	42.0	39.0	-
Not specified	42.0	53.5	-
**Employment status**			KW = 1.58, df = 2, *p* = 0.453
Employed	31.0	33.0	-
Student	36.0	43.0	-
Unemployed	42.0	39.0	-
**Duration of illness (years)**			KW = 5.34, df = 3, *p* = 0.149
< 5	42.0	39.0	-
5–10	32.0	39.0	-
> 10	42.0	53.0	-
Unknown	40.0	42.0	-
**Number of previous admissions**			KW = 8.37, df = 3, *p* = 0.009
First admission	49.0	47.5	-
Two or more	42.0	37.5	-
Unknown	31.0	34.0	-
**Schizophrenia symptom domains**			KW = 1.26, df = 3, *p* = 0.739
Positive	41.0	27.5	-
Negative	32.0	65.0	-
Mixed	42.0	32.0	-
Catatonia	55.0	64.0	-
**Discharge plan**			*U* = 335, *p* = 0.103
Home	42.0	28.0	-
Placement and/or Non-governmental organisation	45.0	141.0	-
**Comorbid psychiatric illness**			*U* = 1482.0, *p* = 0.284
Any psychiatric comorbidity	43.0	35.0	-
No psychiatric comorbidity	42.0	33.0	-
**Substance use pattern**			*U* = 631.5, *p* = 0.333
Single-substance use	40.0	32.0	-
Polysubstance use	44.0	35.0	-
**Type of substance used**			KW = 1.90, df = 3, *p* = 0.593
Alcohol	42.0	41.0	-
Cannabis	42.0	40.0	-
Opioid	49.0	55.0	-
Stimulant	49.0	41.0	-
**Comorbid physical illness**			*U* = 1534.0, *p* = 0.410
Any comorbidity	44.0	43.5	-
No comorbidity	42.0	28.0	-
**Oral antipsychotics**			KW = 40.20, df = 5, *p* < 0.001
Olanzapine	42.0	41.0	-
Amisulpride	56.0	50.0	-
Clozapine	89.0	136.0	-
Haloperidol	17.0	18.0	-
Quetiapine	27.0	33.0	-
Risperidone	43.0	32.0	-
**LAI antipsychotics**			-
Zuclopenthixol decanoate	32.0	25.0	-
Flupentixol decanoate	43.0	70.0	-
**Oral and LAI antipsychotic treatment combination**			KW = 3.84, df = 3, *p* = 0.280
LAI only	55.5	26.5	-
clozapine and LAI	113.5	52.8	-
Non-clozapine AP and LAI	37.0	33.0	-
**Other psychotropic medication**			
Mood stabilisers	44.5	39.5	-
Antidepressants	44.5	32.8	-
Anticholinergics	48.5	71.5	-

U, Mann–Whitney U test U; KW, Kruskal–Wallis test; *df*, degrees of freedom; LAI, long-acting injectable; AP, antipsychotics; IQR, interquartile range.

In the GLM (Table 4), only living alone was associated with a significantly shorter LOS (β = –1.22, *p* = 0.004), corresponding to a 70% reduction in LOS (exp(β) = 0.30, 95% CI: 0.13–0.68). Clozapine use was associated with a 43% longer LOS (exp(β) = 1.43, 95% CI: 0.85–2.38), although this result was not statistically significant (*p* = 0.174) (Table 3).

**TABLE 4:** Generalised linear model results predicting length of stay.

Predictor	Coefficient	s.e.	*Z*-value	*p*-value	95% CI
Intercept	3.62	0.34	10.51	< 0.001	2.95 to 4.30
Living arrangements	−1.22	0.43	−2.86	0.004	−2.05 to 0.38
Admissions	−0.10	0.17	−0.62	0.537	−0.44 to 0.23
Clozapine	0.36	0.26	1.36	0.174	−0.16 to 0.87
Age	0.01	0.01	2.00	0.045	0.00 to 0.03
Gender	−0.12	0.20	−0.60	0.547	−0.51 to 0.27

CI, confidence interval; s.e., standard error.

## Discussion

This study found a median LOS of 42 days, with a mean of 55 days for patients with schizophrenia admitted to CHBAH and discharged either from CHBAH or TARA hospital. This LOS was found to be longer than that reported for other general hospital units internationally. Studies conducted in general hospital units in Canada,^17^ Australia,^14^ Nigeria^18^ and Ethiopia^19^ reported a mean LOS of 20 days, 15 days, 28 days and 32 days, respectively, for patients with schizophrenia. In the African counterparts, system-level differences played a role. For instance, in Nigeria, where the majority of patients covered medical expenses themselves, clinicians were pressured to discharge early to reduce costs. In addition, prior to the implementation of new mental health laws in 2023, Nigeria’s national health insurance scheme had capped hospitalisation to a 15-day limit, resulting in shorter LOS.^18^ Similarly, in Ethiopia, limited psychiatric facilities, the lack of psychiatrically skilled staff and limited social services created increasing pressure for early discharges, which resulted in shorter LOS.^19^

In our study setting, the longer LOS was therefore likely multifactorial, including poorly resourced community psychiatric services for schizophrenia in SA,^5^ as well as the clinical complexities of presentations. We noted that patients presenting for their first psychiatric admission (index admission) in our study had a longer LOS, necessary in order to achieve symptom remission. Other reasons included social factors as discharges were delayed whilst awaiting placement from TARA hospital. In contrast, some patients due to bed pressures were discharged home from CHBAH to await placement in residential care facilities from there.

According to the literature, the LOS for patients with schizophrenia is influenced by many factors, including patient socio-demographic profiles, illness severity, study setting, country-specific mental health legislation, social support, comorbid substance abuse, history of electroconvulsive therapy, restraint use, number of previous admissions and availability of community psychiatric services.^4,14,19,20^

Information from SA is limited, and the only comparable study from Gauteng was conducted at a regional hospital psychiatric unit which limits direct comparison. Nevertheless, the mean LOS in this study is more than double that reported at the regional general hospital unit (19.5 days).^9^ This likely confirms that patients with more illness severity, social needs and clinical needs were possibly admitted to central compared to regional hospitals. The mean LOS in this study for patients who were discharged from TARA hospital (173 days) is also longer than that reported previously for patients with schizophrenia and schizoaffective disorder at a specialised psychiatric hospital in Gauteng (128 days).^15^ A likely explanation for this is that the study did not include the LOS at the general hospital from which patients were transferred.

Living alone compared to living with family was associated with a statistically significantly shorter LOS (*p* = 0.004). It is possible that patients who lived alone were more independent, had milder symptoms and were less functionally impaired than those patients who lived with their families. Supporting this is a study in a high-income country suggesting that early discharge is often facilitated when patients have stable independent living arrangements, as longer LOS risked housing and job losses.^21^ In contrast, an African study found that patients with a diagnosis of schizophrenia without caregiver support had longer LOS,^22^ highlighting context-specific differences.

Living circumstances significantly influence LOS, especially in African societies where family support is the primary form of social care, when other resources are limited.^22^ However, findings are mixed on the influence of caregiver presence. Some studies report that the presence of caregivers was associated with longer LOS, possibly due to caregiver burden and thus using inpatient care for respite whilst ensuring patient safety.^21^ It is also important to note that caregivers may positively influence recovery, reducing LOS.^22^

The demographic profile of patients in our study was consistent with existing literature, with more males admitted for schizophrenia compared to females in SA.^9^ In this study, gender had no significant influence on the LOS, in keeping with a study from the United States^8^ and Malawi.^20^ On the other hand, there is evidence for women having a longer LOS internationally^23,24^ and in SA.^9^

It has been suggested that married patients with supportive partners have a shorter LOS.^23,24^ A study from Ethiopia found that family visits in the hospital were associated with earlier discharges.^19^ However, married patients did not have a significantly shorter LOS in our study. A likely explanation is that our study was not sufficiently powered to detect a difference between married and single patients, given the disproportionate number of single patients (93.2%) compared to married patients (5.3%).

Being on clozapine was associated with a longer LOS in our study, although not statistically significant in regression analysis. This finding was comparable with another SA study,^15^ where longer LOS was possibly attributed to the severity and complexities of patient profiles on clozapine. This is not surprising, given that clozapine is prescribed for treatment-resistant schizophrenia, which was previously found to be associated with higher costs, longer LOS and poorer outcomes.^25^

The study by Goga et al.^15^ highlighted delays in time to clozapine initiation. This was similar in our study where potential candidates, although a relatively small sample for clozapine initiation, were offered alternative antipsychotics. Initiating clozapine prescriptions requires regular laboratory monitoring,^26^ which is challenging, especially in SA’s poorly resourced community psychiatric clinics.^5^ It is likely that the patients were hospitalised during this period because of practical difficulties for patients to get to the clinic for weekly monitoring.

Whilst several studies have attempted to determine the superiority of LAI antipsychotics over oral antipsychotics, findings have not been generalisable.^27^ There is evidence that the prescription of LAI-antipsychotics reduces both relapse and hospitalisation rates, resulting in lower costs of care.^28^ However, in this study, LOS for patients on LAI-antipsychotics was not statistically significant as compared to those patients on oral agents, which may be explained by the underutilisation of LAI-APs.

Although comorbid substance use disorders were common in this study, with most patients using cannabis, whose use is associated with more severe illness,^10^ we found no association between substance use and LOS. This is in keeping with a local study that reported no association between LOS and substance use.^15^ However, another older study at a general hospital psychiatric unit in Gauteng found a shorter LOS in patients with comorbid substance use disorder.^29^ These differences are likely a reflection of patient profiles, service pressure and study setting.

### Limitations

As this was a retrospective study, we examined patient records, some of which had missing or incomplete information. Owing to the format of the discharge summaries, only specifically recorded socio-demographic factors were explored. We did not examine data on readmissions, which may have been valuable in identifying risk factors for relapse, interadmission treatment efficacy and longer-term outcomes overall.

In addition, the statistical analysis was limited due to insufficient sample sizes for certain variables, making it difficult to draw definite conclusions. Length of stay may reflect different practice patterns, availability of resources, and population characteristics depending on the healthcare setting. This variability therefore limits the generalisability of our results.

## Conclusion and recommendations

Our study examined the LOS of patients diagnosed with schizophrenia in an urban, public sector healthcare setting in SA. We found that the LOS in our setting was longer than that reported in other studies in SA. Living alone was associated with a shorter LOS, suggesting that functional independence may facilitate earlier discharge. Actively engaging patients in the development of feasible treatment plans may support the transition to living independently, thus shortening LOS.

Given the constrained mental healthcare budget and a global move towards deinstitutionalisation, inpatient treatment is a concern. This study highlights that the longer LOS presents a challenge of continued reliance on inpatient services, which is significantly longer than their counterparts in Africa. Therefore, mitigating modifiable contributors to increased LOS, such as psychosocial factors (being dependent on caregivers), preventing multiple relapses requiring admissions, and improving awareness strategies focusing on early detection are important. Efforts in initiating clozapine earlier during inpatient admission by identifying suitable candidates and ensuring staff are adequately trained in the initiation of clozapine and may also assist in reducing LOS and improving outcomes.

This study highlights the burden of schizophrenia in our context, highlighting the need for further studies examining the LOS and cost of illness for schizophrenia in other centres in SA. Such studies would be aimed at identifying cost-effective and clinically effective strategies that will help reduce LOS.

## References

[1] Docrat S, Lund C, Besada D. An evaluation of the health system costs of mental health services and programmes in South Africa. Cape Town: University of Cape Town; 2019.

[2] Paliweni-Zwane TI, Modisane LN, Grobler GP. Factors associated with long hospitalisation for psychotic disorder patients in an acute ward: Tertiary care hospital. S Afr J Psychiat. 2024;30:a2049. 10.4102/sajpsychiatry.v30i0.2019 PMC11079344 38726331

[3] Docrat S, Besada D, Cleary S, Daviaud E, Lund C. Mental health system costs, resources and constraints in South Africa: A national survey. Health Policy Plan. 2019;34(9):706–719. 10.1093/heapol/czz088 31544948 PMC6880339

[4] Cheng P, Wang L, Xu L, Zhou Y, Zhang L, Li W. Factors related to the length of stay for patients with schizophrenia: A retrospective study. Front Psychiatry. 2022;12:818254. 10.3389/fpsyt.2021.818254 35140640 PMC8818940

[5] Sorsdahl K, Petersen I, Myers B, Zingela Z, Lund C, Van der Westhuizen C. A reflection of the current status of the mental healthcare system in South Africa. SSM Ment Health. 2023;4:100247. 10.1016/j.ssmmh.2023.100247

[6] Garbi A, Tiniakos I, Mikelatou Z, Drakatos I. Decrease of hospitalizations and length of hospital stay in patients with schizophrenia spectrum disorders or bipolar disorder treated in a mobile mental health service in Insular Greece. Psych. 2021;3(4):780–791. 10.3390/psych3040049

[7] Lund C, Flisher AJ. Community/hospital indicators in South African public sector mental health services. J Ment Health Policy Econ [serial online]. 2003 [cited 2025 Jan 15];6(4):181–187. Available from: https://pubmed.ncbi.nlm.nih.gov/14713725/14713725

[8] Chen E, Bazargan-Hejazi S, Ani C, et al. Schizophrenia hospitalization in the US 2005–2014: Examination of trends in demographics, length of stay, and cost. Medicine. 2021;100(15):e25206. 10.1097/MD.000000000025206 33847618 PMC8052007

[9] Janse van Rensburg A, Olorunju S. Diagnosis and treatment of schizophrenia in a general hospital based acute psychiatric ward. Afr J Psychiatry. 2010;13(3):204–210. 20957319

[10] Nowbath N, Abdelatif N, Lippi G. Comparing the medication costs of treating patients with schizophrenia who use cannabis with those who do not. S Afr J Psychiat. 2024;30:a2211. 10.4102/sajpsychiatry.v30i0.2211 PMC11079364 38726333

[11] Jack H, Wagner RG, Petersen I, et al. Closing the mental health treatment gap in South Africa: A review of costs and cost-effectiveness. Glob Health Action. 2014;7:23431. 10.3402/gha.v7.23431 24848654 PMC4038770

[12] Badriah F, Abe T, Nabeshima Y, Ikeda K, Kuroda K, Hagihara A. Predicting the length of hospital stay of psychiatry patients using signal detection analysis. Psychiatry Res. 2013;210(3):1211–1218. 10.1016/j.psychres.2013.09.019 24095680

[13] Barruel D, Perozziello A, Lefèvre H, Msellati A, Launay C, Dauriac-Le Masson V. Predictors of the length of stay in psychiatric inpatient units: A retrospective study for the Paris Psychiatry Hospital Group. Front Psychiatry. 2024;15:1463415. 10.3389/fpsyt.2024.1463415 39359856 PMC11445158

[14] Zhang J, Harvey C, Andrew C. Factors associated with length of stay and the risk of readmission in an acute psychiatric inpatient facility: A retrospective study. Aust N Z J Psychiatry. 2011;45(7):578–585. 10.3109/00048674.2011.585452 21718126 PMC3190839

[15] Goga LY, Marais BS. Schizophrenia and schizoaffective disorder: Length of stay and associated factors. S Afr J Psychiat. 2024;30:a2237. 10.4102/sajpsychiatry.v30i0.2237 PMC11079426 38726337

[16] Mendenhall E, Kim AW, Panasci A, et al. A mixed-methods, population-based study of a syndemic in Soweto, South Africa. Nat Hum Behav. 2022;6(1):64–73. 10.1038/s41562-021-01242-1 34949783 PMC8799501

[17] Chen S, Collins A, Anderson K, McKenzie K, Kidd S. Patient characteristics, length of stay, and functional improvement for schizophrenia spectrum disorders: A population study of inpatient are in Ontario 2005 to 2015. Can J Psychiatry. 2017;62(12):854–863. 10.1177/0706743716680167 29194005 PMC5714115

[18] Oladeji BD, Ogundele AT, Dairo M. Determinants of length of stay in the psychiatric wards of the University College Hospital, Ibadan, Nigeria. Afr J Med Med Sci. 2012;41(2):147–152. 23185912

[19] Addisu F, Wondafrash M, Chemali Z, Dejene T, Tesfaye M. Length of stay of psychiatric admissions in a general hospital in Ethiopia: A retrospective study. Int J Ment Health Syst. 2015;9:13. 10.1186/s13033-015-0006-x 25780386 PMC4361196

[20] Barnett BS, Kusunzi V, Magola, et al. Factors associated with long length of stay in an inpatient psychiatric unit in Lilongwe, Malawi. Soc Psychiatry Psychiatr Epidemiol. 2019;54(2):235–242. 10.1007/s00127-018-1611-1 30349960 PMC6586467

[21] Clibbens N, Harrop D, Blackett S. Early discharge in acute mental health: A rapid literature review. Int J Ment Health Nurs. 2018;27(5):1305–1325. 10.1111/inm.12515 29949227

[22] Kaggwa MM, Najjuka MS, Kesande C, et al. Length of stay of hospitalized patients at tertiary psychiatry facilities in Uganda: The role of caregiver’s presence. Discov Ment Health. 2022;2(1):15. 10.1007/s44192-022-00018-x 37861871 PMC10501016

[23] Gopalakrishna G, Ithman M, Malwitz K. Predictors of length of stay in a psychiatric hospital. Int J Psychiatry Clin Pract. 2015;19(4):238–244. 10.3109/13651501.2015.1062522 26073671

[24] Tulloch AD, Fearon P, David AS. Length of stay of general psychiatric inpatients in the United States: Systematic review. Adm Policy Ment Health. 2011;38(3):155–168. 10.1007/s10488-010-0310-3 20924662

[25] Kennedy JL, Altar CA, Taylor DL, Degtiar I, Hornberger JC. The social and economic burden of treatment-resistant schizophrenia: A systematic literature review. Int Clin Psychopharmacol. 2014;29(2):63–76. 10.1097/YIC.0b013e32836508e6 23995856

[26] Daniels MV, Ramlall S. Clozapine monitoring at a specialised psychiatric hospital: A retrospective chart review. South Afr J Psychiat. 2023;29:a2039. 10.4102/sajpsychiatry.v29i0.2039 PMC10623633 37928939

[27] Kishimoto T, Robenzadeh A, Leucht C, et al. Long-acting injectable vs oral antipsychotics for relapse prevention in schizophrenia: A meta-analysis of randomized trials. Schizophr Bull. 2014;40(1):192–213. 10.1093/schbul/sbs150 23256986 PMC3885289

[28] Pilon D, Muser E, Lefebvre P, Kamstra R, Emond B, Joshi K. Adherence, healthcare resource utilization and Medicaid spending associated with once monthly paliperidone palmitate versus oral atypical antipsychotic treatment among adults recently diagnosed with schizophrenia. BMC Psychiatry. 2017;17(1):207. 10.1186/s12888-017-1358-3 28576133 PMC5457548

[29] Moosa MYH, Jeenah FY. An analysis of acute admissions to a general hospital psychiatric unit. S Afr Psychiatry Rev. 2002;5:16–18.

